# The Influence of Sex and Hormones on Organelle Stress in Kidney Injury: Insights from Preclinical Models

**DOI:** 10.3390/biology15020173

**Published:** 2026-01-17

**Authors:** Hector Salazar-Gonzalez, Yanet Karina Gutierrez-Mercado, Raquel Echavarria

**Affiliations:** 1 Departamento de Procesos Tecnológicos e Industriales, Instituto Tecnologico y de Estudios Superiores de Occidente (ITESO), Tlaquepaque 45604, Mexico; hector.salazar@iteso.mx; 2 Departamento de Clinicas, Centro Universitario de los Altos, Universidad de Guadalajara, Tepatitlan de Morelos 47620, Mexico; yanet.gutierrez@academicos.udg.mx; 3 Investigadora por Mexico, Secretaria de Ciencia, Humanidades, Tecnologia e Innovacion (SECIHTI), Mexico City 03940, Mexico; 4 Departamento de Ciencias de la Salud, Centro Universitario de los Altos, Universidad de Guadalajara, Tepatitlan de Morelos 47620, Mexico

**Keywords:** organelles, stress, kidney, CKD, sex, hormones, mitochondria, endoplasmic reticulum, cilia

## Abstract

Kidney cells are exposed to a wide range of physiological and pathological stresses, including hormonal changes, mechanical forces, hypoxia, hyperglycemia, and inflammation. These insults can trigger adaptive responses, but when they persist, they can lead to organelle stress. Organelles such as mitochondria, the endoplasmic reticulum, and primary cilia sustain cellular metabolism and tissue homeostasis. When organelle stress occurs, it disrupts cellular processes and organelle communication, leading to metabolic dysfunction, inflammation, fibrosis, and progression of kidney disease. Sex and hormonal factors play a significant role in the development of renal disorders. Many glomerular diseases show distinct differences between the sexes. Chronic Kidney Disease is more common in women, while men often experience a faster decline in kidney function, partly due to the influence of androgens. Additionally, the loss of female hormonal protection after menopause highlights the importance of sex as a factor in renal susceptibility. This narrative review synthesizes preclinical evidence on how sexual dimorphism and sex hormones affect organelle stress in mitochondria, the endoplasmic reticulum, and primary cilia, from 33 studies identified through a non-systematic literature search of the PubMed database, to provide an overview of how these mechanisms contribute to sex-specific differences in kidney disease pathophysiology.

## Simple Summary

Kidney disorders are influenced by many factors, but biological sex and hormones play important roles in many diseases that affect the kidney, showing clear differences between men and women. Chronic Kidney Disease, for example, is more common in women, yet men often lose kidney function faster, partly because male hormones can worsen disease progression. After menopause, women lose some of the protective effects of female hormones, which further highlights how sex influences vulnerability to kidney problems. Kidney cells face many kinds of everyday and disease-related stress. At first, the cells can adjust to these challenges, but if the stress continues, it can overwhelm important parts of the cell. Organelles are cell structures such as mitochondria, the cell’s energy center; the endoplasmic reticulum, which helps make and fold proteins; and primary cilia, small cellular sensors. Organelles work together to maintain cell metabolism and kidney function, but when they are stressed, the cell’s normal activities and communication break down, leading to problems such as reduced energy, inflammation, and scarring. This is a review of animal studies that describe how sex and hormones affect stress in cellular organelles and how these differences may help explain why men and women may experience kidney disease differently.

## 1. Introduction

The kidneys eliminate waste products, maintain electrolyte balance, regulate acid-base homeostasis, control blood pressure, and secrete hormones. Specialized cells within the nephron, the functional unit of mammalian kidneys composed of a glomerulus and a tubule, perform these functions [1]. Within the glomerulus, endothelial cells and podocytes collaborate to regulate the selectivity of the filtration barrier and preserve the integrity of the glomerular basement membrane [2]. Epithelial cells in distinct segments of the renal tubule modulate urine composition and volume [3]. Additional cell types, such as mesangial, interstitial, and immune cells, also contribute to overall kidney function.

Kidney cells experience various physiological and pathological stimuli throughout their lifespan, including hormonal fluctuations, mechanical forces, starvation, high osmolarity, hypoxia, inflammation, hyperglycemia, and molecular damage. While these stimuli can prompt adaptive cellular responses, when they are severe or persistent, they can result in organelle stress [4]. Organelles are specialized structures within cells that are surrounded by membranes, enabling the compartmentalization of biochemical processes vital for energy metabolism, biosynthesis, and homeostasis [5]. Organelles found in kidney cells include mitochondria, the endoplasmic reticulum (ER), primary cilia, the Golgi apparatus, peroxisomes, and lysosomes. These organelles interact closely with one another at membrane contact sites or through intracellular vesicles, exchanging signaling molecules, proteins, lipids, ions, and RNAs. In disease states, organelles may experience stress, disrupting their interactions [6]. This dysregulation can lead to metabolic imbalances, inflammation, and fibrosis, all of which contribute to the progression of kidney diseases [7].

More than 850 million individuals worldwide are estimated to be affected by some form of kidney disease [8]. Kidney disease encompasses a heterogeneous group of disorders that impair renal structure and function in which even minor abnormalities are associated with increased risks of systemic complications and mortality [9]. Chronic kidney disease (CKD) is defined as a long-term condition in which kidney abnormalities persist for at least three months, while acute kidney injury (AKI) is characterized by a rapid decline in kidney function, indicated by a significant rise in serum creatinine or a marked decrease in urine output [10,11]. Diabetes substantially elevates the risk and severity of both AKI and CKD [12]. Furthermore, episodes of AKI in individuals with diabetes often lead to maladaptive repair, accelerated CKD progression, and end-stage renal disease.

Genetics, epigenetics, the immune system, environmental factors, and comorbidities influence the development and progression of kidney disease [13]. Sex and hormones also play a significant role [14]. Many inherited kidney diseases result from mutations in genes located on the X chromosome and predominantly affect males due to the presence of a single active X chromosome. However, the roles of X-chromosome inactivation and the manifestation of kidney phenotypes in females with X-linked kidney disorders, such as Alport syndrome, Lowe syndrome, Fabry disease, Dent disease, and nephrogenic diabetes insipidus are receiving increasing attention [15].

AKI is a significant risk factor for developing CKD [16]. Sexual dimorphism is seen in the progression from AKI to CKD, with males more often advancing to end-stage renal disease [17]. CKD prevalence increases with age, rising from lower rates among young adults to much higher rates in those aged 70 and above [18,19]. Although CKD is more prevalent in women, men experience a faster decline in renal function and are more susceptible to risk factors such as albuminuria and elevated glucose levels [20,21]. The progression of various kidney diseases, including polycystic kidney disease, IgA nephropathy, and membranous glomerulonephritis, is generally slower in women than in men [22]. However, women often start dialysis at a more advanced stage of kidney impairment, have less access to kidney transplantation, and spend longer on waiting lists, even though more than half of kidney donors are women [23,24].

The role of female sex hormones, especially estrogen, in kidney diseases is complex. The protective advantage against CKD progression observed in women decreases after menopause, highlighting the significance of female sex hormones [25]. Evidence indicates an association between increased CKD risk and early menopause, while the use of oral estrogens as hormonal therapy has been associated with reduced renal function in postmenopausal women [26,27,28]. Additionally, studies also report a more rapid decline in kidney function during puberty, coinciding with rising sex hormone levels [29]. Meanwhile, androgens have been shown to contribute to the development of hypertension and related kidney damage in males [14].

Preclinical studies are critical for understanding sex-specific mechanisms in kidney disease and for improving the translational relevance of findings to develop more precise and equitable therapies. Hence, in this narrative review, we examine evidence from preclinical studies on how sexual dimorphism and sex hormones affect organelle stress and contribute to differences in the pathophysiology of renal disease between sexes.

Previous studies have reviewed organelle stress pathways and sex differences in kidney disease [6,7,30]. This narrative review introduces a new framework by examining organelle stress through sexual dimorphism, highlighting the importance of sex as a biological variable in preclinical models to clarify clinically observed sex-specific phenotypes. We aimed to identify studies addressing the roles of biological sex, sex hormones, and organelle stress mechanisms in preclinical models of renal disease. Relevant articles were identified through a search of the PubMed database using the keywords “kidney”, “renal”, “organelle stress”, “mitochondria”, “endoplasmic reticulum”, “cilia”, “sex”, and “sexual dimorphism”. Human studies were excluded by applying the “other animals” filter. Eligible articles were restricted to English-language publications with no date limitations through November 2025. Studies were selected if they included animals of both sexes or interventions targeting sex hormones, reported mechanisms associated with organelle stress in mitochondria, ER, or cilia, and used any experimental model of kidney injury.

This narrative review initially presents mechanistic evidence of organelle stress in kidney injury across each cellular compartment (mitochondria, ER, and cilia) based on the 33 preclinical studies identified (Supplementary File S1). Subsequently, it discusses organelle crosstalk, methodological considerations, existing knowledge gaps, translational implications, future research directions, and conclusions.

## 2. Organelle Stress in Kidney Injury

### 2.1. Mitochondria

The kidneys have one of the highest metabolic rates and contain many mitochondria to support their energy requirements for filtration and reabsorption [31]. AKI, diabetic nephropathy, and glomerular disease are associated with mitochondrial dysfunction, uncoupling of the respiratory chain, oxidative stress, mitochondrial swelling, apoptotic signaling, and cell death [17,32,33]. Sex-dependent differences exist in the structure and function of renal mitochondria [34]. Male kidneys possess larger mitochondria with greater oxidative capacity, whereas female kidneys exhibit lower oxygen consumption and enhanced antioxidant responses. Furthermore, sex hormones modulate mitochondrial function, thereby influencing both physiological and pathological processes in the kidneys.

The ovaries produce estrogen and progesterone, which are sex hormones that regulate the female reproductive cycle. Estrogen acts by binding to estrogen receptor alpha (ERα), estrogen receptor beta (ERβ), and G protein-coupled estrogen receptor 1 (GPER) [35]. All three estrogen receptors (ERα, ERβ, and GPER) are expressed in kidney cells and contribute to renal protection by modulating redox signaling, enhancing mitochondrial respiratory chain complex assembly and activity, increasing ATP synthesis, and defending against oxidative stress [34]. Progesterone has demonstrated similar effects on mitochondrial function [36]. However, not all evidence supports a renoprotective role for estrogen. In rat models of glomerulosclerosis and renal failure associated with hyperlipidemia, hyperinsulinemia, and obesity, ovariectomy reduces glomerulosclerosis, whereas exogenous estrogen accelerates glomerular damage and increases albuminuria [37,38]. The involvement of mitochondrial stress in these adverse effects of estrogen has yet to be determined.

Testosterone is a steroid hormone that regulates the development of male sexual characteristics and exerts its effects through the androgen receptor (AR), which can translocate into mitochondria [39]. The AR modulates the expression of mitochondrial DNA (mtDNA)-encoded genes, decreases the activity of oxidative phosphorylation (OXPHOS) enzymes, and facilitates communication between mitochondria and the nucleus [40]. Dihydrotestosterone alters the energy metabolism of tubular epithelial cells by regulating enzymes involved in glycolysis and β-oxidation in the context of diabetic nephropathy [41]. While the role of testosterone in kidney function is multifaceted, evidence from human and preclinical studies indicates that it may have detrimental effects and contribute to sex-dependent differences in CKD progression [42].

Additionally, pharmacological interventions with metformin, liraglutide, rapamycin, and raloxifene exhibit sex-specific effects on renal mitochondrial stress, underscoring the need for preclinical studies that examine sex- and hormone-related influences on therapeutic responses [34,43,44,45]. Furthermore, pharmacological interventions used in diabetic kidney injury, such as metformin, mineralocorticoid receptor antagonists, and glucagon-like peptide-1 receptor agonists, interact with or are modulated by sex hormone signaling pathways [46,47,48]. These are critical considerations for both research and clinical practice, as these interactions could lead to sex-specific differences in mitochondrial stress activation, affecting drug efficacy and clinical outcomes.

**Figure 1:**
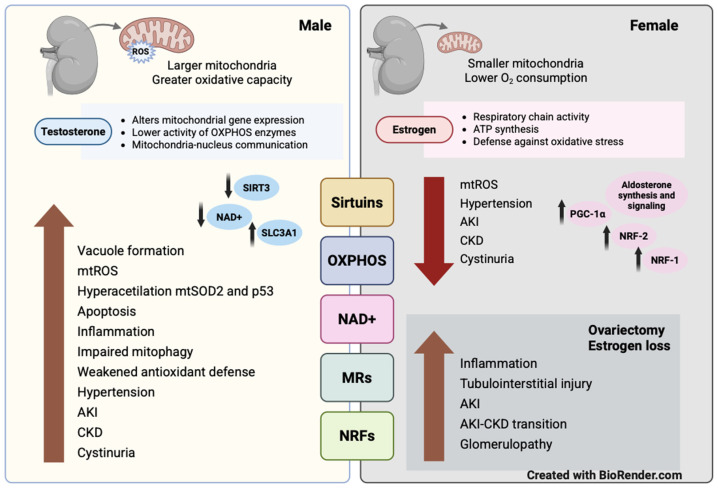
Sex-dimorphic regulators of mitochondrial stress in the kidney.

Multiple mitochondrial stress mechanisms associated with renal disease have been identified in animal models of ischemia–reperfusion, diabetic nephropathy, obstructive nephropathy, and IgA glomerulopathy. However, the influence of sexual dimorphism on mitochondrial stress pathways in the kidney has received less attention. In this section, we discuss evidence on mitochondrial regulators, including sirtuins, OXPHOS components, NAD+ metabolism, mineralocorticoid receptors and aldosterone, NRF-1 and NRF-2, and oxidative status, which are influenced by sex and hormonal factors (Figure 1).

#### 2.1.1. Sirtuins

Sirtuins are a family of conserved mammalian NAD-dependent deacetylases that includes seven members (SIRT1-SIRT7) [49]. Evidence suggests that sex-specific dysregulation of SIRT1, SIRT2, and SIRT3 in AKI and diabetic nephropathy models affects renal metabolism and stress responses [50,51,52,53,54]. SIRT1 and SIRT2 localize to the nucleus and cytoplasm [55,56]. In contrast, SIRT3 localizes to mitochondria, where it deacetylates metabolic enzymes involved in the citric acid cycle, the electron transport chain, the urea cycle, the catabolism of specific amino acids, and the β-oxidation of fatty acids [57,58,59]. SIRT3 activity is required to maintain mitochondrial function and thereby protects against renal injury.

Downregulation of SIRT3 correlates with disease progression in preclinical models of AKI induced by ischemia–reperfusion injury (IRI), cisplatin, and sepsis [60]. Inactivation of SIRT3 in both sexes results in hyperacetylation of mitochondrial superoxide dismutase (SOD2) and p53 within renal tubular epithelial cells, which increases oxidative stress and apoptosis, thereby worsening AKI [61]. *Sirt3*^−^/^−^ knockout mice exhibit elevated oxidative stress, vascular inflammation, and age-dependent hypertension [62]. Restoration of SIRT3 activity reverses these pathological changes. Resveratrol also restores SIRT3 activity, decreases acetylated SOD2 levels, and reduces oxidative stress and mitochondrial damage in renal tubular cells during sepsis-induced AKI [63]. In addition, in a rat model of adenine-induced chronic kidney disease (CKD), peroxisome proliferator-activated receptor-γ coactivator-1α (PGC-1α) protects against vascular calcification by restoring SIRT3 expression in the calcifying aorta, which reduces mitochondrial reactive oxygen species (mtROS) [64]. This observation is clinically significant for CKD patients, as vascular calcification substantially increases the risk of cardiovascular mortality.

Murine models of IRI-induced AKI-to-CKD transition demonstrate that males are more susceptible to injury and subsequent CKD development than females. This disparity is primarily attributed to the influence of sex hormones, as oophorectomized females also develop CKD [17,65,66]. In males, SIRT3 deficiency exacerbates fibrosis following IRI by disrupting mitochondrial dynamics in renal tubular epithelial cells [67]. Differential renal SIRT3 expression accounts for the observed sexual dimorphism in both unilateral and bilateral IRI models of AKI [68]. In males, reduced SIRT3 levels are associated with vacuole formation, increased mtROS, and tubular injury after IRI. Conversely, females display higher SIRT3 levels, which are linked to estrogen-mediated protection of the tubular epithelium. Overexpression of SIRT3 in males provides protection against kidney IRI, while ovariectomized females and female inducible kidney tubule-specific SIRT3 knockdown mice exhibit diminished kidney function and survival after IRI, mirroring the phenotype observed in control males.

#### 2.1.2. OXPHOS

Mitochondrial OXPHOS generates ATP, which supports the energy-intensive transport and reabsorption processes necessary for renal homeostasis [31]. In diabetic nephropathy and IRI, impaired ATP production is especially harmful to proximal tubule epithelial cells due to their substantial energy requirements [69,70].

Sex-specific differences in hemodynamic responses and renal mitochondrial bioenergetics are seen in the Dahl salt-sensitive rat model of hypertension [71,72]. In males, renal cortical mitochondria consume more oxygen and generate more ATP than in females, suggesting higher susceptibility to oxidative injury. Conversely, females have more protective mechanisms against oxidative damage.

Cystinuria, a prevalent inherited cause of kidney stones, affects males more frequently than females [73]. The solute carrier family 3 member 1 (SLC3A1) gene encodes a type II membrane glycoprotein responsible for renal tubular transport of neutral and basic amino acids. Research involving SLC3A1-deficient mice demonstrates that male SLC3A1^−^/^−^ mice develop more severe cystinuria and experience increased oxidative stress, primarily attributed to abnormal mitochondrial morphology and function [74]. Furthermore, kidneys from male *Slc3a1*^−^/^−^ mice exhibit negative enrichment of mitochondrial pathways, decreased expression of OXPHOS-related genes, and reduced ATP levels compared to those of female knockout mice.

Exercise training has been shown to benefit individuals with CKD by enhancing physical function, reducing blood pressure, and improving glycemic control [75]. In a study evaluating hemodynamic parameters and renal mitochondrial bioenergetics in both ovariectomized and intact rats, either sedentary or subjected to resistance training, estrogen deficiency significantly reduced OXPHOS and electron transfer capacity. Additionally, estrogen deficiency decreased the mRNA levels of respiratory complex I and IV subunits in the cortical mitochondria of sedentary animals [76]. These reductions were not observed in rats that participated in resistance training, underscoring the protective effects of both estrogen and exercise on mitochondrial function.

#### 2.1.3. NAD+

NAD+ functions as a coenzyme that is critical for mitochondrial activity and fatty acid oxidation [77]. Renal NAD+ metabolism exhibits sex-specific differences, primarily resulting from the effects of estrogen and androgens. Reduced NAD+ levels are linked to impaired mitophagy, dysregulation of the unfolded protein response (UPR), and diminished antioxidant defenses, all of which contribute to the pathogenesis of AKI and CKD [78,79,80,81].

NAD+ supplementation restores mitochondrial function in preclinical models of Alport syndrome, CKD, and AKI [82,83,84]. PGC-1α protects renal tubular cells against AKI by enhancing transcription of genes involved in NAD+ synthesis. This effect is also seen with NAD+ supplementation [85]. Nicotinamide riboside, a NAD+ precursor, promotes peroxisome proliferator-activated receptor alpha signaling and restores fatty acid oxidation in renal proximal tubules, providing protection to both sexes in a model of Alport syndrome [82]. Nevertheless, males and females show different regulation of transcriptional pathways related to fibrosis and inflammation in this model.

The cystine transporter SLC3A1 is highly expressed in the proximal tubules of male kidneys, likely due to increased mitochondrial activity required to maintain baseline physiological function compared to female kidneys [74]. As a result, male kidneys exhibit greater vulnerability to cystinuria in the absence of SLC3A1. SLC3A1 regulates mitochondrial function by modulating NAD+ uptake, and in cystinuria, reduced NAD+ levels in tubular cells contribute to mitochondrial dysfunction. In preclinical models of cystinuria, supplementation with the NAD+ precursor nicotinamide mononucleotide (NMN) increased mitochondrial NAD+ levels and reduced kidney injury and fibrosis in male *Slc3a1*^−^/^−^ mice. These results suggest that NMN and NAD+ supplementation may effectively address sex-specific differences associated with SLC3A1 deficiency.

#### 2.1.4. MRs and Aldosterone

MRs are directly associated with mitochondrial stress, hypertension, and diabetic nephropathy [86,87,88]. Notably, the nonsteroidal MR antagonist finerenone protects obese, insulin-resistant mice from kidney injury by preventing albuminuria, inflammation, fibrosis, and mitochondrial dysfunction [89].

MRs are nuclear receptors and ligand-dependent transcription factors broadly expressed in kidney cells, particularly in the distal nephron [90]. Aldosterone is the primary agonist of MRs and predominantly targets epithelial sodium channels, thereby promoting sodium and water reabsorption to maintain sodium balance, fluid homeostasis, and blood pressure [91]. Dysregulation of the MR/aldosterone system can result in hypertension. In addition, MRs modulate insulin signaling in various kidney cell types [92,93]. Overactivation of MRs in podocytes, through both aldosterone-dependent and -independent mechanisms, disrupts glomerular function, induces podocyte injury, and contributes to the progression of diabetic nephropathy [88].

Sex and hormonal changes across life stages influence the activity of MRs, aldosterone production, and blood pressure regulation [94]. MRs bind aldosterone, progesterone, and cortisol. In certain tissues, the enzyme 11β-HSD2 inactivates cortisol, so aldosterone becomes the main activator of MRs and maintains blood pressure and fluid balance. Sex steroids affect aldosterone synthesis and signaling. Estradiol increases aldosterone production, while progesterone inhibits aldosterone synthase and competes with aldosterone for binding to MRs. This regulation of aldosterone by sex hormones is linked to hypertension in postmenopausal women [95]. MR agonists worsen renal injury, and MR antagonists are protective in hypertensive male rats [96,97,98]. Renal damage in female rats given aldosterone-salt treatment is also influenced by sex hormones [99].

#### 2.1.5. NRF-1 and NRF-2

NRF-1 and NRF-2 regulate antioxidant defense mechanisms [100]. NRF-1 is upregulated by IRI as part of the macrophage response to oxidative stress, promoting mitochondrial adaptations that reduce inflammation and protect tissue from damage [101]. In contrast, NRF-2 maintains mitochondrial homeostasis and provides protective, time-dependent effects in AKI [102].

Both estrogen receptors, ERα and ERβ, are localized within mitochondria, but their direct impact on mtDNA transcription remains unclear [103]. Estrogen contributes to mitochondrial homeostasis and biogenesis by promoting the nuclear translocation of PGC-1α. Activation of PGC-1α subsequently stimulates NRF-1 and NRF-2, which are essential for the transcription of nuclear-encoded respiratory chain components and mtDNA-specific transcription factors [104]. In murine models, PGC-1α deficiency correlates with increased susceptibility to AKI in females, which could suggest that estrogen-mediated regulation of PGC-1α may protect renal cells from mitochondrial stress [105].

#### 2.1.6. Oxidative Status

Mitochondrial oxidative status reflects the balance between ROS production and antioxidant capacity [106]. Excess ROS damages DNA and proteins and promotes lipid peroxidation, all contributing to kidney injury. Mitochondrial management of oxidative stress differs between sexes. Generally, females display higher mitochondrial oxidative capacity and stronger antioxidant defenses, partly due to sex hormones [107].

Proteinuria exacerbates tubulointerstitial injury and serves as an independent risk factor for CKD progression [108]. The impact of raloxifene, a selective estrogen receptor modulator, was assessed in ICR-derived glomerulonephritis mice, a strain predisposed to nephrotic syndrome [109]. In this proteinuria-induced kidney injury model, ovariectomy in female mice activated the NLRP3 inflammasome in tubular cells, increased cytokine levels, and decreased the expression of β-oxidation genes. Raloxifene administration reversed these alterations, highlighting the role of estrogen in attenuating inflammation, ROS production, and inflammasome assembly in proteinuria-induced kidney injury [110]. The intracellular abundance of ERα seems to determine the extent of estrogen’s effects, while the cellular redox state regulates ERα transcription and its DNA-binding capacity [111]. With advancing age, decreased estrogen or ERα levels reduce anti-inflammatory protection and elevate oxidative stress by impairing mitochondrial function. In aging mouse glomeruli and mesangial cells, both ERα expression and its transcriptional activity decline, which may contribute to the diminished renoprotective effects observed in older females [112].

### 2.2. Endoplasmic Reticulum

The ER is the cell’s largest organelle, made up of interconnected sacs and tubules that form a dynamic network separated from the cytoplasm [113]. It is the main site for protein synthesis, folding, modification, and transport. The ER also handles lipid production, calcium storage, and signaling. Impaired ER homeostasis involves disruptions in the UPR, protein degradation, autophagy, and organelle crosstalk, which can lead to the onset and progression of kidney disease [114,115,116,117].

Elevated markers of ER stress have been identified in the glomeruli and tubular interstitium in preclinical models of kidney disease. Podocytes exhibit vulnerability because of their substantial protein-folding requirements and heightened metabolic activity [118]. Hyperglycemia, chronic proteinuria, dyslipidemia, and impaired insulin signaling are among the factors that can induce ER stress and subsequently trigger podocyte apoptosis.

Prolonged ER stress driven by albuminuria has been reported in podocytes of proteinuric rats and in experimental models of glomerular disease [119,120]. ER stress also contributes to tubular injury in AKI, as shown by the protection in C/EBP homologous protein (CHOP) deficient mice and in mice treated with 4-phenylbutyric acid against tunicamycin-induced AKI [121,122]. In aged female kidneys, exogenous supplementation with diallyl trisulfide, a hydrogen sulfide donor, reduces ER stress, inflammation, and fibrosis induced by IRI [123]. Activation of the ER stress-regulated ATF4/p16 pathway, triggered by elevated deposition of advanced glycation end products, promotes premature senescence of renal tubular epithelial cells during progression of diabetic nephropathy [124].

Sex-specific differences exist in renal responses to ER stress, with female kidneys demonstrating greater resistance to damage [125]. In experimental models, male mice exposed to tunicamycin, a widely used ER stress inducer, show a more pronounced increase in BiP, a member of the heat shock protein 70 family, and spliced X-box binding protein 1 (XBP-1) compared to females. Despite the adaptive protective roles of BiP and XBP-1, male mice exhibit increased susceptibility to ER stress-induced AKI, which is likely attributable to sex-specific variations in ER-mediated apoptosis. Testosterone appears to be a key factor underlying this heightened vulnerability in males. Conversely, estrogen and progesterone generally confer protective effects by maintaining ER homeostasis and potentially reducing ER stress [126,127]. Nevertheless, data on the precise roles of female sex hormones in preclinical models of kidney disease remain limited.

**Figure 2:**
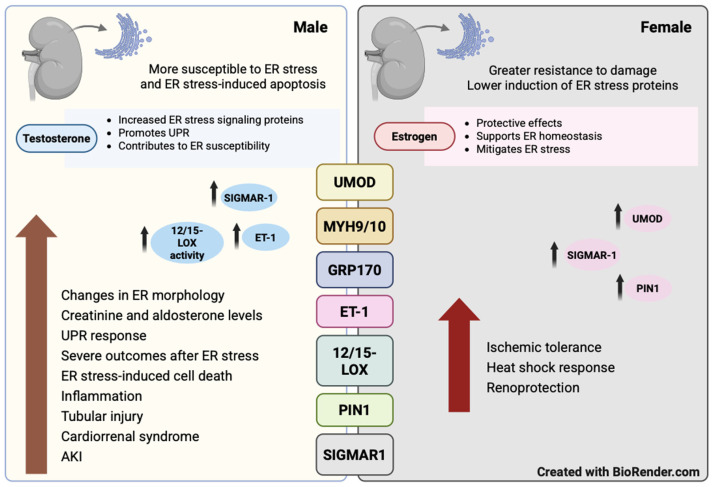
ER stress in kidney injury and its modulation by sex and hormones.

Key ER stress–related mediators influenced by sex and hormones in preclinical models of kidney disease include non-muscle myosin II (NM2) motor proteins, uromodulin (UMOD), glucose-regulated protein 170 (GRP170), endothelin-1 (ET-1), 12/15-lipoxygenase (12/15-LOX), peptidyl-prolyl cis/trans isomerase NIMA-interacting 1 (PIN1), and the Sigma-1 receptor (SIGMAR1) (Figure 2). The following sections examine their respective contributions to renal disease.

#### 2.2.1. NM2 Motor Proteins and UMOD

Myh9 and Myh10 encode the non-muscle myosin heavy chains IIA and IIB, respectively. Mutations in Myh9 are linked to MYH9-related diseases, which affect the blood, eyes, ears, and kidneys [128]. Patients with these mutations may develop progressive proteinuria, glomerulosclerosis, and renal failure. The NM2 motor proteins MYH9 and MYH10 are found in tubular segments, glomeruli, and mesangial cells [129]. Thus, MYH9 and MYH10 in the kidneys support epithelial receptor-mediated transport and maintain podocyte foot processes.

MYH9 and MYH10 facilitate epithelial transport in the kidney by regulating thick ascending limb (TAL)-associated proteins, such as UMOD and the Na^+^-K^+^-2Cl^−^ cotransporter (NKCC2) [130]. Loss of NM2 motor proteins in the renal tubules of adult mice leads to expansion of ER tubules and UMOD accumulation in TAL cells, which induces ER stress, activates the UPR, disrupts ER chaperone expression, and accelerates kidney disease progression. Myh9/Myh10 knockout mice exhibit sex-specific compensatory mechanisms; females are more susceptible to NKCC2 loss and impaired TAL function. Male mice survive approximately twice as long as females due to more effective adaptation of the distal nephron and collecting duct to TAL dysfunction and reduced NKCC2 levels [131]. Both sexes upregulate epithelial sodium channels in medullary collecting ducts, resulting in hypernatremia, but males initially counteract this effect by expressing higher levels of sodium chloride cotransporter.

UMOD-associated kidney disease results from gene mutations that cause misfolded UMOD to accumulate in the ER of distal kidney epithelial cells, rather than being secreted into the urine. Mice carrying a mutation in the *Umod* gene develop progressive kidney disease, driven by the activation of protein kinase RNA-like ER kinase (PERK)/ATF4, an ER stress pathway [132]. Transcriptional profiling of transgenic mouse kidneys expressing the mutant UmodC147W allele demonstrates early upregulation of inflammation and fibrosis, as well as downregulation of lipid metabolism, prior to the onset of functional or histological kidney injury [133]. Evidence suggests that females exhibit higher urinary UMOD levels than males, likely due to estrogen-responsive regulation of the *Umod* gene, which could have important implications for ER stress modulation by female sex hormones [134,135].

#### 2.2.2. GRP170

GRP170 is an ER-resident member of the glucose-regulated protein family that acts as a chaperone, facilitating the folding, assembly, and trafficking of secretory and membrane proteins [136]. Its expression increases during the UPR, a pathway that maintains endoplasmic reticulum proteostasis but may trigger cell death under chronic activation. The UPR is associated with several kidney disorders, including IRI, diabetic nephropathy, and glomerulonephritis [137,138,139]. GRP170 is also critical for mouse embryonic development, and its deletion in kidney nephrons leads to renal failure [140].

Renal epithelial cells need proper ion channel and transporter function to maintain fluid and electrolyte homeostasis. GRP170 is essential for renal proteostasis. Tubule-specific GRP170-deficient mice show severe weight loss, electrolyte wasting, UPR activation, and AKI [141]. Loss of GRP170 also alters ER morphology, with male knockout mice showing higher creatinine and aldosterone levels than females.

UPR induction contributes to hyponatremia and volume depletion in GRP170-deficient mice [142]. Sodium supplementation partially improves electrolyte balance and kidney injury markers in a sex-specific manner; however, it does not restore tubular integrity. These findings support the involvement of UPR activation in the nephron-specific GRP170 knockout phenotype. GRP170 is essential for maintaining ER homeostasis and electrolyte balance. Female mice exhibit a milder phenotype than males, likely due to reduced susceptibility to AKI and a less pronounced UPR.

#### 2.2.3. ET-1

ET-1 is a 21–amino acid peptide with potent vasoconstrictive properties [143]. In the kidneys, nearly all cell types produce ET-1 and express endothelin type A (ETAR) and type B (ETBR) receptors, though endothelial and tubular cells are the main sources of ET-1 [144]. The nephron is rich in ETBR, especially in the TAL and collecting ducts, while ETAR is less abundant. Elevated ET-1 levels occur in hypertension, diabetes, and renal inflammatory pathologies [145]. Excessive or dysregulated ET-1 signaling can contribute to kidney disease. Overexpression of human ET-1 in mice induces renal cyst formation and fibrosis, while ET-1 antagonists have been shown to ameliorate CKD progression [146,147].

The ET-1/ETAR pathway promotes renal ER stress, which initially serves a protective function but may result in apoptotic cell death if prolonged. ET-1 and its receptors modulate tunicamycin-induced ER stress, apoptosis, and organ injury [148]. Activation of ETAR is associated with detrimental effects, while ETBR activation confers protection. Therefore, selective targeting of ETAR represents a potential strategy to reduce ER stress-related kidney injury.

ET-1 signaling is sexually dimorphic in the regulation of blood pressure and sodium handling [149,150,151,152,153]. In models of ischemic AKI, renal ET-1 mRNA levels are significantly higher in male rats compared to females, who fail to show early deleterious changes after ischemia. ETA receptor blockade enhances survival in males but increases mortality in females [154]. However, the specific roles of sex and sex hormones in ET-1–mediated modulation of ER stress in preclinical models of kidney disease have yet to be fully elucidated.

#### 2.2.4. 12/15-LOX

12/15-LOX are iron-containing enzymes that metabolize polyunsaturated fatty acids, converting arachidonic acid into hydroxyeicosatetraenoic acids (HETEs). Dysregulated 12/15-LOX expression and activity contribute to kidney disease development and progression [155,156]. Elevated 12/15-LOX activity is linked to poorer post-ischemic AKI recovery in males compared with females [157]. Male spontaneously hypertensive rats show delayed renal recovery after ischemic AKI. These animals also have higher 12/15-LOX activity, increased 12-HETE levels, ER stress, lipid peroxidation, inflammation, and cell death. Treatment with the 12/15-LOX inhibitor ML355 reduced 12-HETE levels, ER stress, tubular injury, and inflammation, improving renal recovery in males. In females, ML355 had little effect on plasma creatinine but decreased tubular injury and cell death. Persistent 12/15-LOX activation impairs renal recovery in both sexes through ER stress–related mechanisms, with males experiencing more severe outcomes.

A novel link has been proposed between progesterone receptor signaling and 12/15-LOX–mediated fatty acid metabolism in the mouse uterus. Progesterone stimulates the production of 12/15-LOX–derived lipid mediators, which activate peroxisome proliferator-activated receptor gamma and its downstream gene networks, regulating preimplantation in mice [158]. It still needs to be investigated whether a similar mechanism occurs in the kidney.

#### 2.2.5. SIGMAR1

SIGMAR1 is a stress-responsive chaperone primarily located at the mitochondria-associated ER membranes (MAMs) from where it can translocate to the cell membrane, mitochondria, and nucleus [159]. It modulates cell signaling through ligand-protein interactions, making it a key pharmacological target. SIGMAR1 agonists and antagonists induce distinct conformations and oligomeric states, thereby altering its interaction with the ER chaperone BiP and activating downstream signaling. SIGMAR1 is expressed in the kidney and heart, where it contributes to organ protection, the response to ischemic injury, myofibroblast activation, and fibrosis [160].

Sex and hormones influence SIGMAR1 function. In mice, SIGMAR1 shows sexual dimorphism in renal IRI, with 17β-estradiol enhancing the heat shock response and protecting the kidney [161]. In endothelial cells, SIGMAR1 mediates estradiol- and progesterone-driven ET-1 release, but not testosterone, suggesting male and female hormones bind SIGMAR1 at distinct sites to produce opposing effects [162]. Obstructive nephropathy increases SIGMAR1 expression in the kidneys and heart, and SIGMAR1 renal levels augment as the damage progresses, regardless of sex [163]. However, chronic activation of SIGMAR1 with the agonist PRE-084 exacerbates cardiac injury and remodeling following renal damage, with a more pronounced effect in males.

### 2.3. Primary Cilium

The primary cilium, a microtubule-based organelle on vertebrate cells, senses extracellular signals that regulate cellular pathways and maintain homeostasis [164]. In the kidney, cilia are mainly on tubular and collecting duct cells, where they contain signaling proteins essential for epithelial function. Defects in cilia cause ciliopathies that impair kidney development and function, leading to cyst formation and acute tubular necrosis [165]. Autosomal dominant and recessive polycystic kidney diseases, ADPKD and ARPKD, are classic ciliopathies caused by mutations in polycystin-1 (*Pkd1*) and -2 (*Pkd2*).

Cilia are involved in epithelial repair, and changes in cilium length have been linked to kidney injury. Cilium elongation, which alters sensory function during repair, occurs in IRI, acute tubular necrosis, and oxidative stress [166]. Pathological kidneys affected by multicystic dysplastic kidney, focal segmental glomerulosclerosis, and congenital nephrotic syndrome also show dysmorphic primary cilia compared with healthy kidneys [167,168]. Abnormalities in ciliary localization and function are associated with cystogenesis. In contrast, ciliary loss from hyperglycemia accelerates cyst formation, inflammation, and renal injury [169].

**Figure 3:**
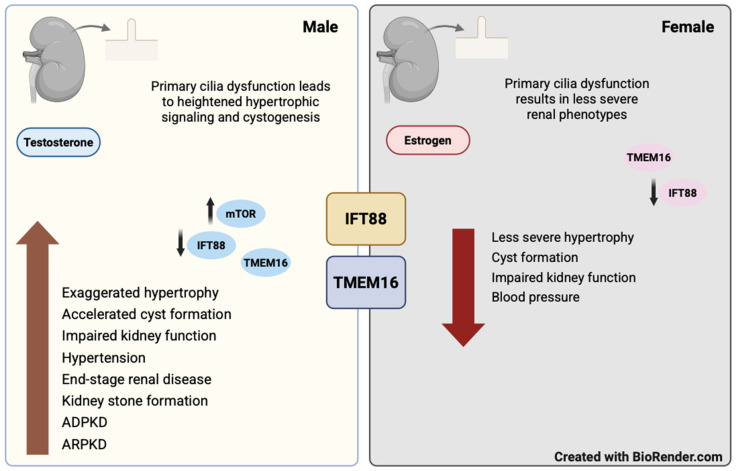
Primary cilia dysfunction and renal disease development in males and females.

Although hundreds of proteins and diverse cellular processes contribute to cilium formation, only a limited number have been linked to sexual dimorphism and regulation by sex hormones. Emerging evidence indicates that intraflagellar transport protein 88 (IFT88) and the calcium-activated chloride channel TMEM16A are key determinants of ciliary function, show sex-dependent regulation, and influence kidney disease (Figure 3).

#### 2.3.1. IFT88

IFT88 is a component of the intraflagellar transport-B complex, which is essential for proper ciliary assembly and transport [170]. Loss of IFT88 disrupts cilium formation, and as a result, animal models harboring *Ift88* mutations are widely used to investigate ciliary function and ciliopathies.

Unilateral nephrectomy induces tubular hypertrophy and proliferation in the remaining kidney, providing a valuable model for investigating how primary cilia regulate renal growth [171]. In both male and female conditional *Ift88* knockouts, cyst onset is delayed [172]. However, following nephrectomy, *Ift88*^−^/^−^ mice exhibit exaggerated hypertrophy, accelerated cyst formation, and impaired kidney function. These mice show elevated mTOR signaling compared with wild-type controls, linking primary cilia dysfunction to heightened hypertrophic signaling and cystogenesis. Consequently, factors that encourage renal hypertrophy may accelerate the progression of adult polycystic kidney disease toward its final stages in both sexes.

Conversely, disruption of IFT88 function in nephron primary cilia produces sex- and age-dependent effects on renal cyst formation, blood pressure, and urinary sodium excretion [173]. Male *Ift88* knockout mice develop hypertension and kidney cysts, whereas females exhibit only modest reductions in blood pressure. In males, elevated blood pressure is associated with sex-specific changes in the expression of nephron sodium transporters and channels.

#### 2.3.2. TMEM16A

TMEM16A localizes to primary cilia, mediates calcium-activated chloride secretion, and promotes proliferation of the cyst-forming epithelium [174]. Loss of TMEM16A expression impairs ciliogenesis and reduces primary cilium length [175]. ADPKD arises from loss of *Pkd1* or *Pkd2* function. Clinically, women with ADPKD typically show slower disease progression than men, likely due to hormonal influences, whereas men have a higher risk of end-stage renal disease and kidney stone formation [176]. TMEM16A plays a central role in ADPKD, as its upregulation enhances intracellular calcium signaling, cell proliferation, and ion secretion [177]. In *Pkd1* knockout mice, males develop a more severe cystic phenotype than females despite comparable TMEM16A expression in renal tubules. Primary renal epithelial cells from males display higher basal intracellular calcium levels, increased proliferation, and larger basal chloride currents, indicating that elevated calcium may enhance TMEM16A activity and thereby promote greater cell proliferation and cyst growth in males.

### 2.4. Organelle Crosstalk

In complex biological systems, organelles do not operate as isolated units but as components of an intercommunicated network. Therefore, a comprehensive understanding of health and disease requires careful consideration of organelle interactions. Increasingly, researchers are recognizing organelle crosstalk as a critical factor in the pathogenesis of kidney disease. Moreover, organelle dysfunction is both a cause and a consequence of kidney injury [6,7]. The maintenance of normal renal function relies on the integrity of cellular organelles, directly compromised by injurious stimuli such as hypoxia and hyperglycemia, while renal dysfunction contributes to organelle stress. This bidirectional damage initiates a self-perpetuating cycle of injury and disease progression. Understanding the nature of these organelle-stress mechanisms is essential for developing novel therapeutic strategies for kidney disease.

Mitochondria are central to cellular calcium homeostasis due to their close interaction with the ER. Disruption of mitochondria–ER crosstalk, particularly at MAMs, contributes to tubular inflammation, fibrosis, and the transition from AKI-to-CKD [6,7]. In diabetic kidney disease, early hemodynamic and metabolic disturbances predispose to irreversible functional and structural damage, with MAMs dysfunction emerging as a key pathological feature [178]. MAMs coordinate critical processes such as glucose metabolism, insulin signaling, lipid handling, ER stress, calcium balance, autophagy, and inflammasome activation. Dysregulation of MAMs impairs mitochondrial quality control, disrupts calcium signaling, and promotes apoptosis, ER stress, and inflammasome activation, all of which exacerbate kidney injury [179].

There is growing evidence of cilia–mitochondria and cilia-ER crosstalk in the development of renal diseases. Primary cilia on renal tubular epithelial cells function as mechanosensors that activate mitochondrial pathways to meet the energy demands of tubular reabsorption [180]. In diabetic kidney disease, abnormal ciliogenesis impairs mitochondrial biogenesis and fatty acid metabolism, ultimately contributing to renal fibrosis [181]. Similarly, after ischemia–reperfusion injury, loss of primary cilia in mice leads to reduced mitochondrial ATP synthase levels and elevated tryptophan levels, further underscoring the importance of ciliary signaling in mitochondrial function [182].

Primary cilia and their associated ion channels are critical for cellular signaling and the maintenance of normal kidney architecture and function [183]. Key ciliary ion channels include transient receptor potential channels, the cystic fibrosis transmembrane conductance regulator, and polycystin 1 (PC1) and 2 (PC2) channels, which are localized both on the plasma membrane and within the ER. PC1 and PC2 interact with the inositol trisphosphate receptor in the ER to regulate intracellular calcium levels. PC1 undergoes autoproteolytic cleavage at its G-protein-coupled receptor proteolytic site within the ER [184]. Meanwhile, PC2 appears to mediate extracellular shear stress-dependent calcium signaling in renal epithelial primary cilia, as well as functioning as an intracellular calcium release channel in the ER [185,186]. Thus, elucidating the specific functions and mechanisms of action of ciliary ion channels and their communication with the ER can provide significant insights into the pathophysiology of renal diseases, particularly polycystic kidney diseases [187].

Despite this evidence, how sex and hormones influence organelle crosstalk during pathological changes in the kidney is still unclear. Understanding these interactions may explain differences in disease presentation, progression, and treatment response between men and women, and among women at different life stages.

## 3. Methodological Considerations and Existing Knowledge Gaps

This work is a narrative review and carries inherent limitations, including subjectivity and potential selection bias, given its flexible, non-exhaustive methodology. This limits reproducibility compared with systematic reviews [188]. Nonetheless, the review followed a clearly defined scope focused on finding evidence of the influence of sex and sex hormones on organelle stress in preclinical models of kidney disease. We sought studies examining the roles of sex, sex hormones, and organelle stress mechanisms involving mitochondria, the ER, and primary cilia in preclinical models of renal disease. Eligible articles were identified in PubMed, restricted to English-language publications with no date limitations through November 2025. Studies were selected if they included animals of both sexes or an intervention affecting sex hormones, reported mechanisms of organelle stress in mitochondria, ER, or cilia, and used any experimental model of kidney injury. The selected literature was organized by organelle and then by pathway.

The present review was not designed to address a specific hypothesis-driven question, but rather to provide an integrative overview of the available evidence on the influence of biological sex and sex hormones on organelle stress in preclinical models of renal disease. Using this approach, we identified 33 studies demonstrating that sex and sex hormones contribute to distinct mechanistic pathways of organelle stress in renal cells across multiple experimental models of kidney injury (Supplementary File S1). These findings are highly relevant for interpreting clinically observed sex-specific phenotypes in human renal disease.

Importantly, this review also reveals a pronounced sex bias in the existing literature, which perpetuates critical knowledge gaps regarding sexually dimorphic mechanisms that influence renal disease incidence and progression. A substantial proportion of preclinical studies fail to incorporate biological sex as an experimental variable, relying predominantly on single-sex models, most often male animals. Consequently, although several mechanisms of organelle stress and inter-organelle crosstalk have been described elsewhere [6,7,30], the modulatory roles of sex and sex hormones on many of these mechanisms remain largely unexplored. Collectively, the evidence synthesized in this review supports a significant role for sex and sex hormones in renal disease progression and organelle stress, while emphasizing the urgent need for preclinical studies to incorporate sex as a biological variable in experimental design and to analyze outcomes with appropriate sex-stratified approaches.

As a limitation, we did not systematically discuss sex-related effects that may vary according to species, strain, age, injury model, or type of hormonal intervention. Furthermore, the mechanistic pathways described should not be considered fully established, as causal evidence remains limited. Also, hormonal cycling in females was not addressed.

## 4. Translational Implications and Future Directions

Epidemiological data indicate that kidney disease prevalence and progression are strongly influenced by biological sex and hormonal changes across the lifespan [33,34,35,36,39,41]. Therefore, preclinical models of renal disease that consider sex as a biological variable are essential for elucidating the molecular mechanisms of organelle stress affecting the kidney, and for developing more effective, personalized therapies to reduce the burden of kidney disease. However, a major barrier to translating preclinical findings into successful treatments is the disconnection between the design of preclinical animal studies and that of human clinical trials [189]. Closer alignment of experimental models with the structure of randomized controlled trials may improve the translational reliability of preclinical research [189]. This includes the use of disease-relevant animal models, assessment of appropriate disease stages, balanced inclusion of both sexes to reflect clinical populations, and selection of clinically meaningful primary endpoints with adequately powered group sizes.

## 5. Conclusions

Organelle stress represents a central mechanism linking persistent cellular stressors to kidney dysfunction and disease progression. Mitochondria, the ER, and primary cilia coordinate to maintain renal homeostasis, and their dysfunction disrupts metabolic, inflammatory, and fibrotic pathways. Sex and hormonal factors influence organelle function under both physiological and stress conditions, contributing to the observed differences in disease susceptibility, progression, and outcomes between males and females.

Here, we identified 33 preclinical studies addressing the role of sex and sex hormones in organelle stress and kidney injury. The principal pathways exhibiting sex-dependent differences were predominantly associated with mitochondrial function, including sirtuins, OXPHOS, NAD+ metabolism, MRs, NRF signaling, and oxidative status. In the endoplasmic reticulum, sex-related effects were reported for NM2 motor proteins, UMOD, GRP170, ET-1, 12/15-LOX, and SIGMAR1. In primary cilia, sex-dependent differences were identified in IFT88- and TMEM16A-associated pathways.

However, many mechanisms underlying organelle stress and inter-organelle crosstalk in renal cells remain poorly understood, particularly when examined through the lens of sexual dimorphism and sex hormone modulation. Hopefully, this narrative review can highlight the importance of conducting preclinical research that includes both sexes and accounts for hormonal fluctuations, as understanding the interplay between organelle stress and sexual dimorphism will provide valuable insights into the pathophysiology of kidney disease and the potential for sex-specific therapeutic strategies aimed at preserving organelle function and mitigating renal injury.

## Data Availability

No new data were created or analyzed in this study. Data sharing is not applicable to this article.

## Supplementary Materials

The following supporting information can be downloaded at: https://www.mdpi.com/article/10.3390/biology15020173/s1. File S1: Identified Preclinical Studies Addressing Organelle Stress in Kidney Injury. References [50,51,54,61,62,63,68,72,74,76,82,99,105,109,112,125,130,131,132,133,141,142,151,152,153,154,157,160,161,163,172,173,177] are cited in the Supplementary Materials.

## Abbreviations

The following abbreviations are used in this manuscript:

12/15-LOX	12/15-lipoxygenase
ATF4	Activating transcription factor 4
AKI	Acute kidney injury
AR	Androgen receptor
ADPKD	Autosomal dominant polycystic kidney disease
ARPKD	Autosomal recessive polycystic kidney disease
CHOP	C/EBP homologous protein
TMEM16A	Calcium-activated chloride channel
CKD	Chronic kidney disease
ET-1	Endothelin 1
ER	Endoplasmic reticulum
ERα	Estrogen receptor alpha
ERβ	Estrogen receptor beta
ETAR	Endothelin type A receptor
ETBR	Endothelin type B receptor
GPER1	G protein-coupled estrogen receptor 1
GRP170	Glucose-regulated protein 170
HETEs	Hydroxyeicosatetraenoic acids
IFT88	Intraflagellar transport protein 88
IRI	Ischemia–reperfusion injury
MRs	Mineralocorticoid receptors
MAMs	Mitochondria-associated ER membranes
mtROS	Mitochondrial reactive oxygen species
mtDNA	Mitochondrial DNA
NAD+	Nicotinamide adenine dinucleotide
NMN	Nicotinamide mononucleotide
NM2	Non-muscle myosin II
MYH9	Non-muscle myosin heavy chains IIA
MYH10	Non-muscle myosin heavy chains IIB
NRF-1	Nuclear factor erythroid 1
NRF-2	Nuclear factor erythroid 2
OXPHOS	Oxidative phosphorylation
PIN1	Peptidyl-prolyl cis/trans isomerase NIMA-interacting 1
PGC-1α	Peroxisome proliferator-activated receptor-γ coactivator-1α
PKD1/PC1	Polycystin 1
PKD2/PC2	Polycystin 2
PERK	Protein kinase RNA-like ER kinase
SIGMAR1	Sigma-1 receptor
SIRT1	Sirtuin 1
SIRT2	Sirtuin 2
SIRT3	Sirtuin 3
SLC3A1	Solute carrier family 3 member 1
SOD2	Superoxide dismutase 2
NKCC2	Na^+^-K^+^-2Cl^−^ cotransporter
TAL	Thick ascending limb
UPR	Unfolded protein response
UMOD	Uromodulin
XBP-1	X-box binding protein 1
